# Use of Automated Irrigating Drainage System in Six Patients With Chronic Subdural Hematoma: A Single-Center Experience

**DOI:** 10.7759/cureus.17355

**Published:** 2021-08-21

**Authors:** Jordan Davies, Alexander S Himstead, Ji Hyun Kim, Alvin Y Chan, Diem Kieu Tran, Frank P Hsu, Sumeet Vadera

**Affiliations:** 1 Department of Neurological Surgery, University of California Irvine, Irvine, USA; 2 Department of Neuroscience, Johns Hopkins University, Baltimore, USA

**Keywords:** irrigating subdural drain, chronic subdural hematoma evacuation, automated irrigating drainage system, chronic subdural hematoma, craniotomy, irraflow, length of stay, pneumocephalus

## Abstract

Background

Chronic subdural hematoma (cSDH) is predicted to become the most common intracranial neurosurgical condition by 2030. Recurrence is estimated between 5-15%, and the use of a surgical drain is associated with lower recurrence rates. The authors present their experience with six patients undergoing cSDH evacuation with an irrigating drainage system, comprising the largest single-institution group in the United States (US).

Methods

IRB-approved, retrospective chart review was performed for six patients who underwent irrigating surgical drain placement during cSDH evacuation. Outcome measures included device settings and duration of the irrigating drain, postoperative length of stay, neurological status at follow-up, and hematoma recurrence.

Results

There were no recurrences noted within this group at last follow-up, with an average follow-up length over three months. The average postoperative length of stay was 2.67 ± 0.51 days. Patients were drained on average for 1.41 ± 0.49 days at 0cm water, irrigating at 55.25 ± 46.44cc/hr. On postoperative day one, average hematoma size and midline shift (MLS) reduction were respectively 13.43 ± 3.31mm and 5.71 ± 1.33mm. No device-related complications were noted.

Conclusion

The authors' early experience with this irrigating drainage device demonstrates that it is safe and effective for this population. Although this is a preliminary study on a small sample size, the excellent results warrant further investigation and establishment of a standard protocol to compare against current treatment regimens.

## Introduction

Subdural hematoma is common in the elderly population partly due to increased use of anticoagulants, decreased brain volume, and increased fall risk [1,2]. Common symptoms include headache, seizures, gait abnormalities, memory deficits, or weakness. Chronic subdural hematoma (cSDH) is a well-studied neurosurgical pathology with many surgical treatment techniques, including surgical evacuation, needle decompression, subdural port evacuation, and endovascular embolization [3-5]. cSDH is predicted to become the most common cranial surgical condition by 2030 with approximately 60,000 annual cases [6]. Recurrence rate is estimated at around 10%, with higher rates associated with anticoagulant use, brain atrophy, hematoma thickness, bilateral disease, baseline comorbidities, post-evacuation brain compression or pneumocephalus, duration of hematoma presence, and advanced age [7-14]. An increasing body of evidence supports the use of surgical drains after hematoma evacuation as a means to lower recurrence rates [7,15-17].

The IRRAflow® drain (IRRAS, Stockholm, Sweden) is a dual-lumen catheter recently FDA approved in the United States (US) that allows for concurrent irrigation and drainage of fluid from the intracranial space while providing a constant measurement of intracranial pressure (ICP). We previously reported the first use of IRRAflow after craniotomy for cSDH evacuation [18]. The use of an automated irrigating subdural catheter may aid in brain re-expansion by breaking up further clots left after surgery and evacuating postoperative pneumocephalus.

In this study, we provide the first grouped retrospective analysis of the use of an irrigating subdural drain after a craniotomy to treat cSDH in six patients. We highlight trends in our experience and a possible approach to future implementation of this technology and treatment protocol. This represents the largest current collection of such patients in the world.

## Materials and methods

IRB approval was submitted and accepted (University of California, Irvine, Institutional Review Board issued approval HS# 2020-5823) for a retrospective chart review of all patients at our institution who underwent subdural hematoma evacuation between January 2019 and October 2020 with IRRAflow placement. As it was a retrospective chart review, patient consent was not required. There was a minimum of two weeks of follow-up (two weeks to one year), with average follow-up of over three months. Data points included subdural size, magnitude of midline shift (MLS), neurologic preoperative and postoperative outcomes, length of stay (LOS), complications, recurrence, and duration, as well as irrigation and drainage settings for the IRRAflow device.

Intervention

All surgical interventions were performed in the operating room after patients were properly identified. Craniotomies were planned and performed in five of the six patients. One patient had bilateral cSDH, and two burr holes were used on each side. For patients undergoing craniotomy, a single burr hole was created, and a craniotome was used to turn the craniotomy flap. The dura was coagulated and opened in cruciate fashion, and if membranes were encountered, they were fenestrated and coagulated within the visible field. Copious irrigation was used to evacuate the hematoma collection until the irrigation returned clear. If acute blood clots were encountered, they were expelled gently with irrigation and suction. Any visualized bridging veins were cauterized and incised. If an inner membrane was noted beneath the subdural collection, it was also fenestrated and coagulated within the visible field. The IRRAflow irrigating catheter was tunneled towards the incision from a prepped portion of skin in the operative field. It was then placed into the subdural space through the craniotomy defect without a stylet. Once the drain was visualized within the proper cranial compartment (subdural), the craniotomy flap was placed over the defect and fixed to the skull with titanium plates in at least three places. After ensuring the drain exited the cranial compartment through the burr hole, and the course of the drain appeared relaxed, the skin was approximated with absorbable sutures and closed with staples.

For the patient undergoing the burr hole evacuation bilaterally, after the burr holes were created, the dura was coagulated and opened in a cruciate fashion. Irrigation was used to express the subdural collection until it ran clear. The IRRAflow catheters were tunneled as described above and placed in the posterior burr hole on each side. No plating system was used, and the incision was closed as per above.

**Figure 1:**
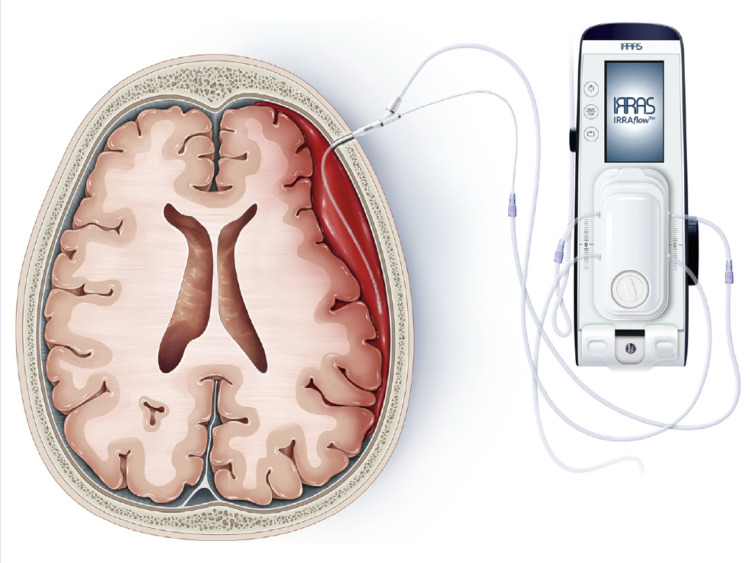
Schematic diagram of IRRAflow® dual-lumen catheter, the only automatic irrigating drain. It can irrigate, drain, and monitor ICP simultaneously. Re-used with permission from: Tran DK, et al. [18] ICP: Increased intracranial pressure

After the IRRAflow catheter is connected postoperatively, there are several settings that can be manipulated. The catheter is capable of irrigating, draining, and measuring ICP. The provider chooses a level to drain at, similar to an external ventricular drain, with 0 being level with the catheter. The irrigation setting can be set to irrigate a certain amount of fluid every 30 or 60 seconds. Alternatively, it can be set to drain-only mode without irrigation. It is imperative to ensure the drainage is at least equal to the amount of irrigation to ensure there is no addition of fluid to the subdural compartment. Figure 1 shows a schematic diagram of the IRRAflow drain and pump inside the subdural space.

## Results

Baseline characteristics and review of the data from six patients who underwent placement of an irrigating subdural catheter after craniotomy for cSDH evacuation are displayed in Table 1 and Table 2, respectively. No symptomatic recurrences were noted. The average postoperative length of stay was 2.67 ± 0.51 days. The average postoperative length of ICU stay was 1.83 ± 0.41 days. Patients were drained for 1.41 ± 0.49 days at the setting of 0, 55.25 ± 46.44cc/hr. The average reduction of hematoma size and MLS at postoperative day (POD) one were respectively 13.43 ± 3.31mm and 5.71 ± 1.33mm.

**Table 1:** Summary of baseline characteristics Further information regarding postoperative size of clot, residual midline shift, length of stay, days in the ICU, days with a drain, recurrence rate, mortality rate, and overall complication rate are reported in table 2. *0 mm MLS occurred in one patient with bilateral SDH MLS: Midline shift; SDH: Subdural hematoma

Summary of baseline characteristics	
Sex	Number (%)
Male	6 (100%)
Female	0 (0%)
Use of anticoagulants	0 (0%)
Use of antiplatelet agents	
Aspirin	2 (33.3%)
Plavix	1 (16.7%)
Age	80.8 (75-89)
Initial size (mm)	26.7 (20-30)
Initial MLS (mm)	7 (0-12)*

**Table 2:** Treatment results Data encompassing treatment results, hospital stay, morbidity, and mortality are provided as mean (range). *Overall postoperative morbidity includes surgical infections, seizures, postoperative bleeds, new neurologic deficits, and misplaced catheters. Morbidity numbers are compared at the time of hospital discharge. †Mortality and recurrence numbers are for all follow-up. MLS: Midline shift.

Treatment Results	IRRAflow
Hematoma size at 24h	13.4 (11.0-20.0)
Change in hematoma size	11.0 (5.0-16.0)
MLS at 24h	5.71 (0.0-7.0)
Change in midline shift	3.14 (3.0-7.0)
Length of stay	2.67 (2.0-4.0)
ICU days	1.83 (1.0-3.0)
Days with surgical drain	1.41 (1.0-3.0)
Morbidity/Mortality	N (%)
Overall postoperative morbidity*	0 (0%)
Mortality^†^	0 (0%)
Recurrence^†^	0 (0%)

**Figure 2:**
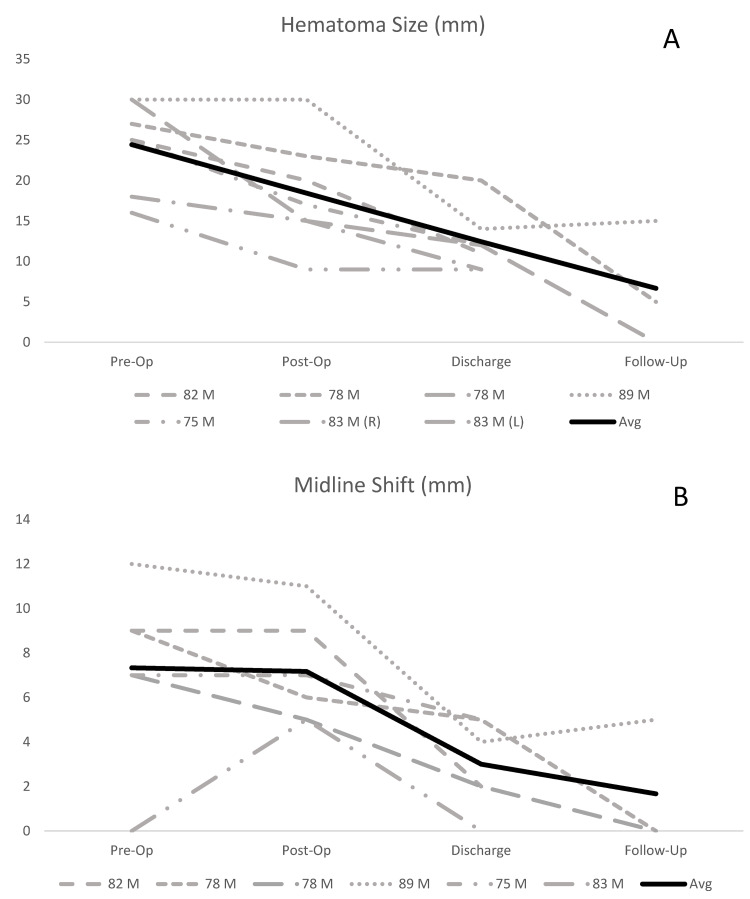
Graphical representation of patient clinical data Trends in hematoma size (A) and MLS (B) in six patients from CT imaging. Three of six patients received imaging after discharge, two at three months and one at nine days. MLS: Midline shift

Individual patient radiographic data are displayed in Table 3 and charted in Figure 2 (A and B). There were no complications in the placement or removal of the irrigating drain, no spontaneous recurrences to date, no infections, and an improved neurologic exam compared to before surgery. One patient (1/6, 16.7%) returned to the emergency department with seizures on POD five and was hospitalized for seizure management. The patient returned to baseline and was discharged without further seizure activity. This is not reflected in Table 2, which displays morbidity/mortality at discharge, and was the only complication in our cohort. Two patients reported one questionable seizure episode each and followed up with neurology but had negative EEG and were taken off anti-epileptic drugs with no further episodes. Given the ambiguity and negative EEG findings, we did not classify these as postoperative seizures. Individual patient clinical outcome is displayed in Table 4.

**Table 3:** Patient clinical data Data regarding presentation, irrigating drainage settings, postoperative examination, discharge disposition, and follow-up examination for each patient. ARU = Acute rehabilitation unit; SNF = Skilled nursing facility; POD: Postoperative day

Patient Clinical Data					
Age/Sex	Symptoms at presentation	Drain settings	Symptoms at 24h postop	Discharge disposition	Symptoms at follow-up
82 M	Right-sided weakness and hemineglect, mental deterioration	Drain at 0, 1.5cc/hr	Full strength, oriented x3	Home	Two weeks: Full strength, mild discoordination and difficulty speaking
78 M	Mild left upper extremity weakness (4+/5), incoordination	Drain at 0, 10cc/hr	Full strength, oriented x3	Home	10 days: L hand paresthesia, full strength. Five weeks: Subjective L hand weakness, on Keppra 500mg Three months: Normal exam, no complaints, continued Keppra One year: Normal exam, no complaints, stopped Keppra
83 M	Mild global weakness, mental deterioration, pupillary asymmetry	Drain at -5, 100cc/hr -> 40cc/hr (POD1)	Equal/reactive pupils. minimal residual weakness	Home	Two weeks: Complete resolution of preoperative neurologic deficits
78 M	Left-sided weakness with pronator drift	Drain at 0, 90cc/hr	Full strength, oriented x3	ARU	Two weeks: no complaints. Normal neurologic exam 3 months: Remains asymptomatic
89 M	Right upper extremity hypertonia, incoordination, mental deterioration	Drain at 0, 30cc/hr	Improved cognition, no formal motor examination	SNF	Five days: Returned to hospital for seizure, improved with medical management Three months: No focal weakness (global 4+/5) Nine months: full strength, no further seizures despite stopping Keppra four months prior
75 M	Mild left upper extremity weakness (4+/5) with pronator drift, incoordination	Drain at 0, 100cc/hr	Minimal residual weakness, no pronator drift	ARU	Two weeks: Complete resolution of preoperative neurologic deficits

**Table 4:** Patient radiographic data Radiographic variables hematoma size and MLS (mm) are reported from CT imaging from each patient at four time points, if available. MLS: Midline shift

Patient Radiographic Data					
Age/Sex	CT variable	Pre-Op	Post-Op	Discharge	Follow-Up
82 M	Hematoma size (mm)	25	20	11	No long-term follow-up imaging obtained
	MLS (mm)	9	9	2	
78 M	Hematoma size (mm)	27	23	20	5 (3 months post-op)
	MLS (mm)	9	6	5	0 (3 months post-op)
83 M	R Hematoma size (mm)	16	9	9	No long-term follow-up imaging obtained
	L Hematoma size (mm)	18	15	9	
	MLS (mm)	0	5	0	
78 M	Hematoma size (mm)	30	15	12	0 (3 months post-op)
	MLS (mm)	7	5	2	0 (3 months post-op)
89 M	Hematoma size (mm)	30	30	14	15 (9 days post-op)
	MLS (mm)	12	11	4	5 (9 days post-op)
75 M	Hematoma size (mm)	25	17	12	No long-term follow-up imaging obtained
	MLS (mm)	7	7	5	

## Discussion

This study represents the largest grouped retrospective case analysis on the utilization of an automated irrigating drainage system following craniotomy for treatment of cSDH in six patients at a single institution. In 2009, Santarius et al. demonstrated that postoperative drainage was associated with a significantly lower rate of cSDH recurrence (9.3% vs. 24%, p=0.003) [19]. However, due to the prevalence of symptomatic hematoma recurrences, neurosurgeons have continued to seek novel means of reducing this risk [20]. One such method is concomitant irrigation, which has been associated with lower recurrence rates in several studies [21-24]. Continuous irrigating drainage has been studied in cSDH as early as 1999, when Hennig and Kloster demonstrated a 2.6% recurrence rate with continuous irrigation and drainage compared to 23.8% with passive drainage (P<0.001) [21]. A case report subsequently illustrated the placement of a reservoir shunt, which was irrigated once daily via insertion of a needle into the shunt chamber and resulted in no hematoma recurrence [23]. Most recently, a cohort study by Sjavik et al. compared continuous irrigation and drainage (using two separate catheters placed in the hematoma cavity) with passive subdural drainage (SDD) (catheter placed in the hematoma cavity set to gravity) and active subgaleal drainage (catheter placed over the burr hole set to suction) [24]. The authors found a lower recurrence rate with continuous irrigating drainage (10.8%) and active subgaleal drainage (11.1%) compared to passive SDD (20.0%, p<0.001) [24]. The authors did report a higher complication rate in the irrigating drainage cohort (14.5% vs. 7.3% in SDD and 8.1% in subgaleal drainage, p=0.019) [24], but this may be attributed to increased risk of microtrauma or brain irritation from the presence of two catheters in the subdural space. While this is a potential drawback of any subdural catheter system, the single dual-lumen catheter featured by the IRRAflow reduces the risk of local microtrauma. Another recent innovation involving continuous irrigating drainage with the IRRAflow was demonstrated in a case report where it was used in conjunction with middle meningeal artery embolization to successfully treat a case of recurrent cSDH [25].

Table 5 displays comparisons to the only recent randomized control trial (RCT) comparing subperiosteal with subdural drains following SDH evacuation [26]. We report a decreased length of stay (2.67 vs. 6.0). Comparison of postoperative morbidity revealed decreased postoperative bleeds, seizures, infection, and misplaced drainage catheters. Overall, we had no postoperative complications in this small sample, compared to 12% in the subperiosteal drainage (SPD) group and 19% in the SDD group. Although hematoma recurrence is a frequent complication of cSDH requiring repeated surgical evacuation [14], there have been no recurrences in our cohort, compared to 8.33% for subperiosteal drainage and 12% for SDD. We also had no misplaced catheters in our cohort, compared to 17% in SDD, which may relate to improved catheter visibility during open craniotomy, compared to the burr hole placement employed by Soleman et al. Our limited sample size and indirect comparison preclude any definitive conclusions. One patient did suffer a postoperative seizure after discharge but had recovery with medical management and no persistent neurologic deficits. Two patients with transient neurologic symptoms concerning seizures followed up with neurology but had negative electroencephalograms and no further episodes in the absence of anti-epileptic medications.

**Table 5:** Data comparing IRRAflow® (treatment results, hospital stay, morbidity, and mortality) with randomized controlled trials utilizing subperiosteal drains (SPD) and subdural drains (SDD) The randomized controlled trials utilizing SPD and SDD used for comparison are from Soleman, et al. [26]. The sample size for IRRAflow is six patients, compared to 120 for SPD and 100 for SDD. *Length of stay in our cohort is listed as the length of stay after surgery, whereas the other data is cited as the length of stay in days, without clarification of the relation to operative time. †Overall postoperative morbidity is defined as surgical infections, seizures, and postoperative bleeds. Morbidity numbers are compared at the time of hospital discharge. ‡Mortality and recurrence numbers are for all follow-up. MLS: Midline shift.

Treatment results	IRRAflow	SPD	SDD
Hematoma size at 24h	13.4 (11.0-20.0)	11.5 (7.7-17.0)	13 (9.0-17.0)
Change in hematoma size	11.0 (5.0-16.0)	11	11.9
MLS at 24h	5.71 (0.0-7.0)	3 (0.0-5.0)	3 (1.0-5.0)
Change in MLS	3.14 (-3.0-7.0)	3 (4.0-5.0)	4 (3.0-5.5)
Length of stay*	2.67 (2.0-4.0)	6 (5.0-9.2)	6 (5.0-8.0)
ICU days	1.83 (1.0-3.0)	Not reported	Not reported
Days with surgical drain	1.41 (1.0-3.0)	2	2
Morbidity/Mortality N (%)			
Post-operative bleed	0 (0%)	10 (8%)	10 (10%)
Seizures	0 (0%)	4 (3%)	5 (5%)
Post-operative infection	0 (0%)	0 (0%)	4 (4%)
New neurologic deficits	0 (0%)	5 (4%)	2 (2%)
Misplaced catheter	0 (0%)	0 (0%)	17 (17%)
Overall postoperative morbidity^†^	0 (0%)	14 (12%)	19 (19%)
Mortality^‡^	0 (0%)	12 (10%)	10 (10%)
Recurrence^‡^	0 (0%)	10 (8.33%)	12 (12%)

In the present study, continuous irrigating drainage was associated with an excellent reduction in hematoma size and MLS, no postoperative bleeding, hematoma recurrence, infection, new neurological deficits, or mortality, and a short length of ICU and hospital stay. The mechanism of continuous irrigating drainage systems in the reduction of hematoma recurrence requires further study. The pathogenesis of cSDH recurrence likely involves collection of inflammatory mediators and fibrinolytic factors in the hematoma cavity [8]. Burr hole evacuation often introduces significant air into the hematoma cavity, resulting in pneumocephalus, which has been associated with delayed cerebral re-expansion [27,28]. This is potentially problematic, as a recently published retrospective review on 291 patients found that persistently depressed brain volume is an independent factor predictive of hematoma recurrence [14]. While manual drain irrigation reduces postoperative pneumocephalus, it can lead to a deleterious increase in intracranial pressure due to the Monro-Keille doctrine, which states that an equilibrium exists between brain parenchyma, blood, and CSF [29]. By this hypothesis, irrigation may confer an increased pressure in the subdural space, which would transfer to the brain parenchyma raising ICP. Irrigating drainage systems are specifically set so that the irrigation rate is gentle yet consistent and is always lower than drainage rate from the cavity. This net negative (drainage > irrigation) drainage theoretically improves brain re-expansion by minimizing postoperative collections - i.e. pneumocephalus, recurrent hematoma, operative bleeding - without increasing ICP. Our results support this hypothesis as the magnitude of MLS decreased substantially by discharge and entirely in both patients with long-term follow-up imaging (Figure 2B).

Limitations

We attempted to compare our cohort with the only randomized controlled trial on subperiosteal and subdural drains by Soleman et al. (Table 5), but there were several limitations. Although it is the largest series of patients treated with an automated irrigating drainage system to date, the sample size is rather small. We report a shorter hospital length of stay compared to the average length of six days reported by Soleman et al [26], but this may not be a fair comparison as the prior study was performed outside the US and different health systems prioritize hospital stay and bed availability unequally. Furthermore, we reported postoperative length of stay, and it is not clear whether Soleman et al. reported postoperative length of stay or total hospital duration. We did not obtain clinical data regarding neurological outcomes (such as the Glasgow Coma Scale (GCS), the modified Rankin Scale (mRS), or the Mackwalder scale), which would provide further information regarding the clinical consequences of utilizing irrigating drainage systems. We reported data as mean (range), while Soleman et al. reported data as median (IQR), a more commonly employed format in Europe. Our POD-one imaging occurred the morning after surgery, typically 12-16 hours post-op, while they reported post-operative imaging data at 24 hours. Follow-up periods were not standardized and were on average shorter than the 12-month follow-up reported by Soleman et al. Given this limitation, we cannot state definitively that hematoma recurrence will not occur in our cohort in the future. However, the excellent postoperative brain re-expansion demonstrated by our cohort is promising. These discrepancies further limit the comparability of our results with their trial. In addition, no patients in our cohort were anticoagulated prior to admission, which may have artificially lowered the risk of hematoma recurrence in our sample.

Other potential drawbacks of the IRRAflow system include local microtrauma from catheter placement within the hematoma cavity, device failure, ICP monitor failure, infection, catheter occlusion, or malposition. Reassuringly, we did not experience these complications in our small sample. Furthermore, these risks are not unique to the IRRAflow and continuous irrigation may lower the risk of infection and catheter occlusion. Despite these limitations, we believe the absence of catheter-related complications and hematoma recurrence, excellent brain re-expansion, unanimous improvement in neurologic deficits, and trend towards a shorter length of stay to represent a promising new avenue for postoperative management of cSDH evacuation. Future directions will include establishing a protocol for follow-up examinations and imaging in this cohort and creating a registry to combine data from several institutions.

## Conclusions

cSDH is a common neurosurgical pathology with disagreement concerning optimal surgical and postoperative management. The mainstay of treatment includes surgical evacuation with burr hole, twist-drill, or mini craniotomy, and subdural, subgaleal, and continuous irrigating drainage as adjunctive means of reducing hematoma recurrence. It is hypothesized that irrigating drainage systems may reduce hematoma recurrence by improving cerebral re-expansion, a known risk factor for recurrence. This study represents the largest review to date on the use of a continuous irrigating surgical drainage system after craniotomy and demonstrates a safe and effective method of treating chronic subdural hematoma. In our series on six patients, we found no catheter-related complications, no hematoma recurrences, excellent brain re-expansion, and improvement in neurological symptoms in each patient. While evidence to support this device remains in its early stages, these preliminary results warrant further investigation to establish the precise role of irrigating surgical drains in the management of CSDH. Future work will establish a standard protocol to compare against current treatment regimens to evaluate the impact on patient outcomes, recurrence, hospital length of stay, and cost.
